# Experimental Study on Dual-Structure Polymer Optical Fiber Sensors for Turbidity Detection

**DOI:** 10.3390/s26020351

**Published:** 2026-01-06

**Authors:** Jiafeng Zhang, Zhibin Liu, Junshi Li, Jiangu Qian, Bing Zhou, Haihua Zhang

**Affiliations:** 1 Shanghai CAAC New Era Airport Design & Research Institute Co., Ltd., Shanghai 200335, China; jiafeng6060@163.com (J.Z.); junshili@163.com (J.L.); zhouzbbing@163.com (B.Z.); 2 Department of Geotechnical Engineering, Tongji University, Shanghai 200092, China; qianjiangu@tongji.edu.cn (J.Q.); haihua_zhang@tongji.edu.cn (H.Z.)

**Keywords:** polymer optical fiber (POF), turbidity monitoring, reflection-refraction type, gap type, particle concentration

## Abstract

This study presents a comprehensive investigation of turbidity monitoring using two different types of polymer optical fiber (POF) sensors: the reflection–refraction type (RR-POF) and the gap type (Gap-POF). Both sensors were used to visualize and monitor the turbidity changes in suspensions with varying concentrations and different particle compositions, namely silica powder and clay particles. The experiments were conducted by introducing silica powder and clay into water at various concentrations, and the resulting turbidity was measured using both types of POF sensors. The results revealed a significant correlation between particle concentration and light intensity for both kinds of POF sensors. As the particle concentration increased, the light intensity decreased due to increased scattering and absorption effects. For both silica powder and clay suspensions, the light intensity stabilized at lower values as the concentration increased, with the Gap-POF sensor exhibiting higher sensitivity to turbidity changes, particularly at high particle concentrations. Additionally, the study found that the particle composition influenced the sensor response. Silica powder particles caused more irregular fluctuations in light intensity at higher concentrations due to their larger particle size and tendency to aggregate, while clay particles, due to their smaller size and better dispersion, resulted in more stable and gradual changes in light intensity. This highlighted the differences in optical responses between different particle types. Furthermore, the multi-wavelength measurements showed consistent results, with white and green lights exhibiting the strongest response to turbidity changes, while red and blue lights were less sensitive. This wavelength-dependent response was attributed to the scattering and absorption properties of the particles in the suspensions. Both RR-POF and Gap-POF sensors proved to be effective for turbidity monitoring, with Gap-POF demonstrating superior performance in high-concentration suspensions. The findings suggest that POF sensors, particularly Gap-POF, are highly suitable for real-time turbidity monitoring in various particle suspension systems.

## 1. Introduction

Turbidity is widely used as an indicator of water quality because suspended particles directly affect the optical transparency of water [1]. It is caused by fine solids such as sand, silt, clay, and organic matter, which scatter and absorb light and thereby reduce water clarity [2,3,4,5,6,7]. Optical instruments are the mainstream approach for turbidity measurement, as turbidity reflects the degree to which suspended particles interfere with light transmission and can be used as an indirect indicator of suspended sediment concentration. Suspended sediment concentration provides important information on erosion processes, sediment transport, and depositional dynamics in aquatic systems [8,9].

Changes in land use can influence hydrological and geomorphological conditions, leading to variations in sediment fluxes and erosion intensity. Optical turbidity sensors have therefore been widely used for high-frequency monitoring, as they provide continuous and quantitative assessments of sediment transport in real time. Once an appropriate calibration curve is established, turbidity can serve as a reliable surrogate for suspended sediment concentration. Previous studies have demonstrated the effectiveness of optical turbidity measurements for sediment monitoring. Skarbøvik et al. [10] used optical sensors to explore the correlation between turbidity and suspended sediment concentration. Gentile et al. [11] reported linear relationships between optical measurements and gravimetric data using immersion probes that simultaneously measured turbidity and suspended sediment concentration. Turbidity has also been applied as a rapid and cost-effective indicator of sediment erosion in urban runoff and construction environments. Memon et al. [12] observed a strong correlation between suspended particles and turbidity levels at construction sites, and Line et al. [13] found that both turbidity and sediment loss increased markedly during highway construction. Xu et al. [14] further demonstrated that automated turbidity-based monitoring provides an effective means for evaluating hillslope erosion processes and that turbidity and spectral characteristics can be used to infer sediment concentrations at relatively low levels. Their findings further showed that turbidity and spectral characteristics can be used to infer sediment concentrations at relatively low levels. On the other hand, excessive suspended particles in water may indicate potential problems such as internal erosion, pipeline cracking, or structural degradation. In drainage systems, variations in surface turbidity often serve as early warning signals of deformation, settlement, loosening, or corrosion within the structure. Consequently, turbidity has been recognized as an important indicator of internal erosion in geotechnical and hydraulic engineering and has received increasing attention from researchers worldwide. Sato and Kuwano [15,16] demonstrated that turbidity in discharge water can effectively indicate the onset and progression of seepage and internal erosion, reflecting the preferential migration of fine soil particles and exhibiting a linear correlation with the amount of eroded material. Ishimaru et al. [17] further revealed, through turbidity-concentration tracking during seepage tests, that finer soil particles tend to remain suspended within pore water rather than transmitting stress.

Despite increasing academic interest, conventional turbidity monitoring still relies heavily on laboratory sampling and single-point sensing. Common analytical techniques include spectrophotometry, visual comparison methods, portable meters, and laboratory turbidimeters [18]. However, laboratory instruments are limited in their ability to measure high turbidity levels and cannot provide continuous or online monitoring. Although these approaches yield useful data, they suffer from inherent drawbacks such as time delays from laboratory analysis and poor adaptability of bulky sensors for long-term or buried applications. Standard in situ turbidimeters have been widely used in water treatment facilities, yet their deployment in natural environments remains challenging. Most commercial devices operate within relatively low turbidity ranges and are unsuitable for high-sediment waters such as rivers, estuaries, and coastal zones [19,20]. High instrument cost, complex calibration, and maintenance requirements further restrict large-scale field application. Moreover, the variability of natural sediment composition introduces additional uncertainty to in situ turbidity measurements. To overcome these limitations, several studies have explored low-cost and efficient turbidity sensing systems [21,22,23,24], which have shown promising performance under controlled laboratory conditions.

To address the limitations of conventional turbidity measurement techniques, polymer optical fiber (POF)-based sensing technologies have attracted growing research interest [25]. These sensors utilize changes in light propagation within the fiber to achieve sensitive, rapid, and stable real-time monitoring under complex environmental conditions. POF sensors also provide advantages such as corrosion resistance, immunity to electromagnetic interference, and strong suitability for long-term in situ deployment [26,27,28,29]. Their low material and fabrication cost further supports their use as a cost-effective and scalable solution for turbidity monitoring in geotechnical and hydraulic engineering. In this study, the feasibility of POF sensors for turbidity detection was systematically evaluated through controlled laboratory experiments. Two configurations were examined: the refractive reflection type (RR POF) and the gap type (Gap POF). These sensors were applied to monitor turbidity variations under different particle concentrations and sediment compositions. By analyzing their optical response characteristics under varying conditions, this research aims to clarify the applicability of POF technology in practical engineering scenarios.

## 2. Sensor and Experimental Procedure

### 2.1. Introduction of POF Sensors

**Figure 1:**
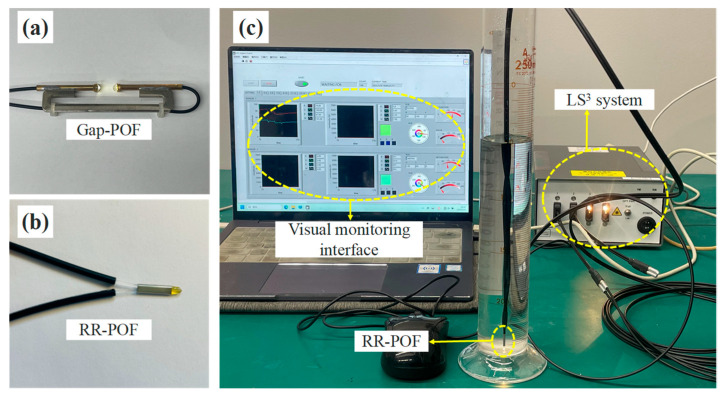
Two types of POF sensors and a layout for turbidity monitoring, (**a**) physical photograph of the Gap-POF sensor, (**b**) physical photograph of the RR-POF sensor, (**c**) actual monitoring interface of the POF optical demodulation system.

As shown in Figure 1, two types of POF sensors with different structural designs and sensing mechanisms were employed in this study. Figure 1a illustrates the Gap-POF, in which two polymer optical fibers are arranged with a 10 mm gap between them. Its monitoring principle is based on the variation in refraction and reflection caused by the different media occupying the gap, which further affects the transmission and reception of optical signals. For instance, when fine particles are present in the gap and obstruct the propagation of light, the received light intensity decays rapidly. In contrast, Figure 1b presents the RR-POF. In this design, the bare ends of two POFs are each cut at 45° and bonded together to form a prism-shaped probe with a 90° tip. One fiber serves as the incident fiber, transmitting light into the prism tip, while the other fiber receives the refracted and reflected light. Based on the principles of refraction and total reflection, the RR-POF exhibits the maximum light intensity in air, whereas the light intensity decreases in pure water. A detailed description of the sensing path of the RR-POF has been provided in our previous work [25]. As shown in Figure 1c, the Light State Sensing System (LS3) was employed to measure the light intensity returned by the POF sensors. The optical data logger is equipped with an LED light source and a data recording unit, which can be operated through dedicated software installed on a personal computer. Since LS3 adopts a 12-bit digital color sensor to detect the three primary colors of visible light (red, green, and blue), the optical signal is output as a 12-bit serial digital signal [25]. The maximum value of each color channel is 4095 (2^12^ − 1), with the initial reading starting from zero. In this study, the white light intensity was defined as the square root of the sum of the squared RGB values, and its maximum light intensity value was 7093.

The experiments employed a broadband white LED as the illumination source, covering the spectral range from approximately 400 nm to 700 nm. The use of a wide-range LED was motivated by the need to evaluate the spectral response characteristics of the POF sensors under different turbidity levels, since light-particle interactions are known to vary with wavelength. Shorter wavelengths in the blue and green regions are more strongly affected by scattering from fine suspended particles, while longer wavelengths in the red region experience relatively weaker scattering and absorption. By employing a broadband LED, the relative spectral responses of the POF sensors can be evaluated simultaneously across the entire visible range without configuring multiple monochromatic light sources. This enables a more comprehensive examination of how turbidity influences the optical signal. Moreover, the broadband LED is cost-effective, easy to integrate with POF-based sensing systems, and supports the research goal of developing a low-cost turbidity monitoring approach.

**Figure 2:**
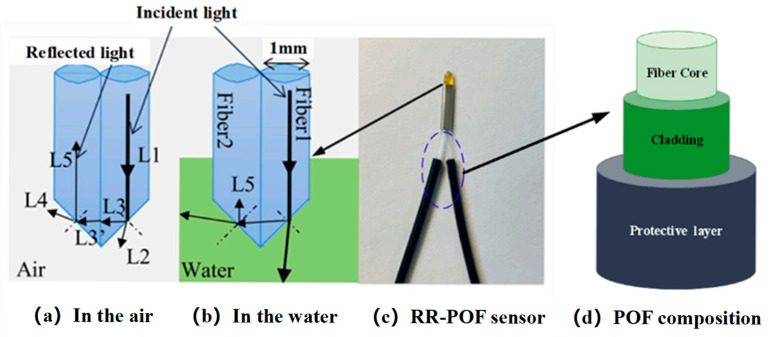
Schematic diagram of light sensor changes in the RR-POF sensor.

The structure and working principle of the RR-POF sensor are illustrated in Figure 2. The prism-shaped sensing tip is formed by polishing two bare polymer fibers at 45° and bonding them together with transparent adhesive to create a right-angle triangular interface. Light emitted from Fiber 1 (L1) reaches the inclined sensing plane, where part of the beam is reflected and part is refracted. A portion of the reflected component (L3′) enters Fiber 2 and then interacts with the second sensing plane, where additional reflection and refraction occur before the output signal (L5) is detected at the end of Fiber 2. The optical paths in air and in water are shown in Figure 2a,b.

The reflected intensity depends on the refractive indices of the POF core and the surrounding medium. The core refractive index of the POF is approximately 1.49, whereas the refractive indices of air and water are about 1.000 and 1.333, respectively. According to the total internal reflection condition. The critical angle is smaller in air than in water. As a result, a larger fraction of light is totally internally reflected at the sensing interface in air, and the intensity of L5 is higher in air than in pure water. When suspended particles are added to the water, the propagation of light is further modified. The particles introduce local refractive index inhomogeneities and cause multiple scattering near the sensing interface. In this configuration, part of the light that would otherwise be transmitted into the bulk water is scattered back into the acceptance cone of Fiber 2 and is partially re-coupled into the reflected path. Therefore, compared with pure water, the presence of fine suspended particles near the sensing tip modifies the local refractive index distribution and introduces additional scattering, which increases the amount of light that is redirected back into Fiber 2 and leads to an enhancement of the reflected intensity L5.

**Figure 3:**
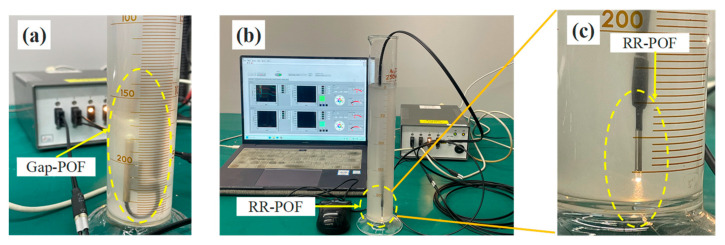
Turbidity monitoring process using two different types of POF sensors, (**a**) turbidity monitoring process using the Gap-POF sensor, (**b**) turbidity monitoring process using the RR-POF sensor, (**c**) enlarged view of the RR-POF monitoring region.

As illustrated in Figure 3, the two structural types of POF sensors were applied in the turbidity monitoring experiments. It can be observed that the presence of fine suspended particles alters the optical processes occurring at the sensing interfaces of the fibers. When particles are located near the sensing tip, local variations in the refractive index are produced within the surrounding medium, and additional scattering events are generated in the region adjacent to the prism surface. These effects influence the reflection and refraction conditions at the inclined interfaces and lead to modifications in the angular distribution, phase composition, and spatial dispersion of the returned light.

Furthermore, the heterogeneous refractive-index field created by suspended particles results in partial redirection of light that would otherwise be transmitted into the water. A portion of this redirected light is guided back toward the acceptance region of the receiving fiber, which increases the effective coupling efficiency of the reflected signal. At the same time, multiple scattering near the sensing interface produces an expansion of the backward-scattered optical component. The combined influence of refractive-index perturbation and particle-induced scattering therefore changes the propagation characteristics of the optical signal within the sensor and produces measurable variations in the detected intensity. These intensity variations are captured in real time and can be visualized through the monitoring interface.

### 2.2. Experimental Materials

**Figure 4:**
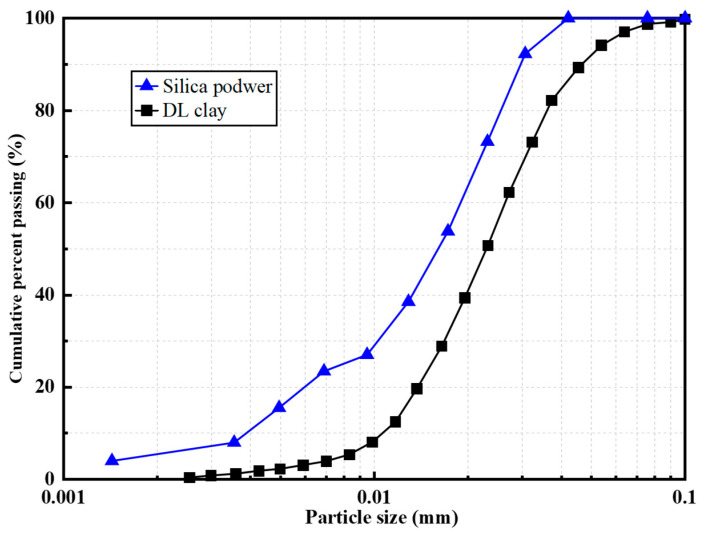
Particle size distribution curves of silica powder and DL clay used in turbidity monitoring.

In this study, silica powder and DL clay particles were selected as the primary constituents of turbidity suspensions. The silica powder consisted of fine particles obtained from crushed silica sand, classified as non-plastic fine-grained soil. DL clay, on the other hand, is commonly adopted in geotechnical laboratory testing. The particle size distribution curves of silica powder and DL clay are shown in Figure 4, which demonstrates that the particle size of silica powder is generally larger than that of DL clay. These two fine-grained soils with different particle sizes and mineral compositions were selected as the primary monitoring objects. Suspensions of varying concentrations were prepared by thoroughly mixing different dry weights of silica powder and DL clay with deionized water, which were subsequently monitored using POF sensors in a visualized manner.

## 3. Experimental Monitoring Results

### 3.1. RR-POF Turbidity Monitoring

#### 3.1.1. Monitoring of Silica Powder Suspensions by RR-POF

**Figure 5:**
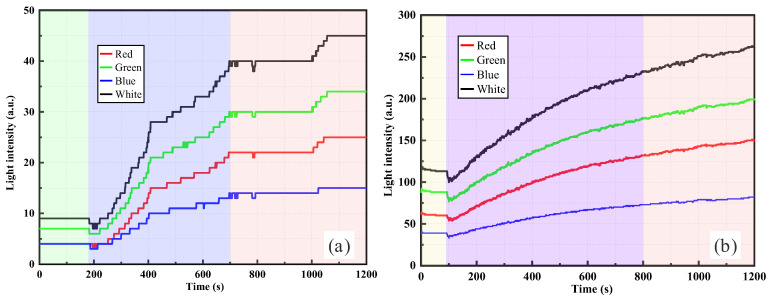
RR-POF light intensity monitoring results in silica powder suspensions: (**a**) 0.5 g dry weight, (**b**) 1.0 g dry weight.

As shown in Figure 5, the temporal evolution of light intensity measured by the RR-POF sensor exhibited significant differences under various concentrations of silica powder suspensions. Figure 5a presents the response curve under the condition of 0.5 g silica powder. A sudden change in light intensity was observed immediately after the suspension was introduced, followed by a stepwise increase. This characteristic indicates that sedimentation fronts or particle aggregates intermittently passed the probe, leading to stagewise increases in local turbidity. In contrast, Figure 5b shows the curve corresponding to 1.0 g of silica powder, where the light intensity rose more rapidly and smoothly, eventually reaching a higher plateau. This suggests that, in higher concentration suspensions, particle flux became more continuous and uniformly distributed, resulting in greater overall turbidity. Multiband measurements demonstrated consistent results, with all curves showing monotonic increases followed by stabilization. Among the wavelengths, green and white light exhibited the strongest responses, while red and blue light were weaker. This behavior is closely related to the wavelength-dependent transmission loss characteristics of polymer optical fibers and differences in the optical source power. Mechanistically, suspended particles induce scattering and multiple scattering of incident light, with part of the scattered light being effectively redirected and re-coupled into the fiber, thereby enhancing the signal intensity with increasing particle concentration.

**Figure 6:**
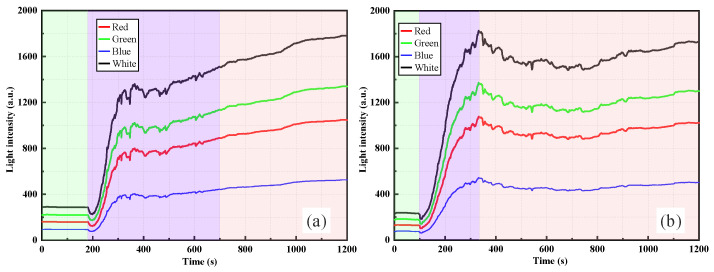
RR-POF light intensity monitoring results in silica powder suspensions: (**a**) 1.5 g dry weight, (**b**) 2.0 g dry weight.

As shown in Figure 6, when the dry weights of silica powder were 1.5 g and 2.0 g, the RR-POF sensor exhibited even more pronounced and significant variations in light intensity. Similar to the lower concentration cases, the light intensity remained at the minimum baseline in pure water and experienced an abrupt change at the instant when the suspension was introduced. In Figure 6a, under the 1.5 g condition, light intensity rose sharply within a short time and then gradually transitioned into a slower growth stage after approximately 200–300 s. The overall curve exhibited a two-phase trend: a rapid initial increase followed by a gradual rise. In contrast, Figure 6b shows the response at 2.0 g, where light intensity increased even more rapidly, reaching a peak within 200–400 s, followed by partial decline and oscillations before gradually stabilizing. This phenomenon suggests that, at higher concentrations, stronger particle–particle interactions occurred, with aggregation, sedimentation, and redistribution introducing greater disturbances to the optical path. Consequently, temporary decreases and fluctuations in light intensity appeared after the initial rise. Multiband monitoring again showed consistent trends, with the order of responses remaining as white > green > red > blue. Among these, the white and green channels exhibited the most pronounced amplitudes, capturing the dynamic evolution of particle concentration and distribution with high sensitivity. Although red and blue channels produced relatively weaker responses, they still followed the same overall trend. From a mechanistic perspective, as the concentration of suspended particles increases, scattering and multiple scattering effects are significantly enhanced, allowing more scattered light to be redirected and re-coupled into the fiber, which leads to a rapid rise in light intensity. However, at excessively high concentrations, competitive scattering and absorption effects, combined with sedimentation processes, cause the signal to fluctuate or decline after the peak, as observed in Figure 6b.

In summary, the comparison between Figure 5 and Figure 6 reveals that, with increasing concentration, the RR-POF sensor response undergoes a clear evolution characterized by “stepwise increase–smooth rise–continuous growth–peak fluctuation.” This progression reflects the transition of suspension systems from low-concentration intermittent particle distributions to high-concentration strong particle interactions, providing critical insights for establishing quantitative relationships between particle concentration and light intensity.

#### 3.1.2. Monitoring of Clay Particle Suspensions by RR-POF

**Figure 7:**
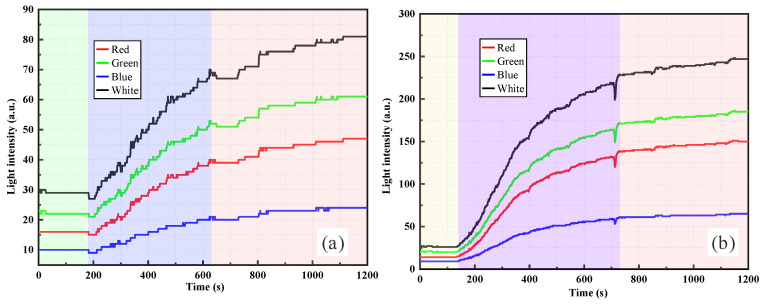
RR-POF light intensity monitoring results in clay suspensions: (**a**) 0.5 g dry weight, (**b**) 1.0 g dry weight.

As shown in Figure 7, the light intensity response of the RR-POF refractive–reflective sensor in clay suspensions exhibited typical temporal evolution patterns, but with certain distinct differences compared with the silica powder system. Figure 7a shows the curve under the condition of 0.5 g of clay. During the pure water stage, the light intensity remained at a low level. At the instant the clay suspension was introduced, a sudden jump in light intensity occurred, followed by a gradual increase that presented a stepwise enhancement. This phenomenon is similar to that observed in low-concentration silica suspensions, indicating that at low dosages, clay particles also intermittently passed the probe in the form of aggregates or sedimentation fronts, leading to stagewise increments in the signal. Figure 7b illustrates the response at 1.0 g clay, where light intensity rose rapidly after injection and presented a smooth quasi-exponential curve, ultimately reaching a higher plateau. This suggests that at higher concentrations, clay particles are dispersed more uniformly, resulting in more continuous fluxes and significantly enhanced turbidity, with a more stable signal evolution process. Compared with silica powder, clay particles—due to their smaller particle size and larger specific surface area—more readily form stable colloidal dispersions when suspended in water. Therefore, under the same mass condition, the amplitude of light intensity response was relatively greater, and the curve tended to be smoother. Multiband results again showed consistency, with light intensity ranking as white > green > red > blue. Among these, white and green light were the most sensitive to turbidity variations, while red and blue exhibited weaker signals. This trend is closely related to the wavelength-dependent loss characteristics of polymer optical fibers and the spectral power distribution of the light source. Mechanistically, dispersed clay particles induce scattering and multiple scattering of incident light, with part of the scattered light effectively redirected and re-coupled into the fiber. As particle concentration increased, scattering and redistribution effects were progressively enhanced, causing light intensity to monotonically rise over time before eventually stabilizing.

**Figure 8:**
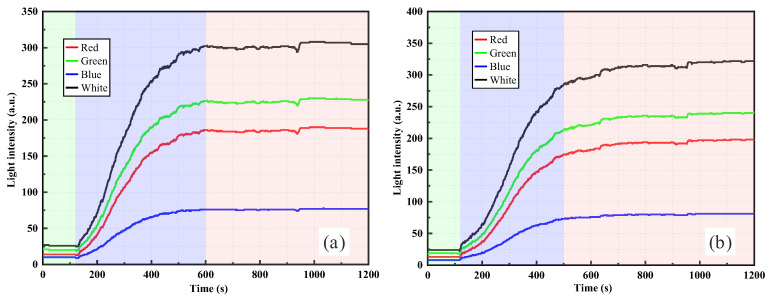
RR-POF light intensity monitoring results in clay suspensions: (**a**) 1.5 g dry weight, (**b**) 2.0 g dry weight.

Figure 8 presents the RR-POF monitoring results for clay suspensions with dry weights of 1.5 g and 2.0 g. Compared with the low-concentration stages, high-concentration suspensions exhibited faster rising rates and higher stable platforms. In Figure 8a, under the 1.5 g condition, light intensity increased sharply after suspension injection and reached stability around 300–400 s, with a relatively smooth overall curve and a stable plateau phase. In Figure 8b, under the 2.0 g condition, the response amplitude further increased, the initial rise was more rapid, and the plateau value was significantly higher than that of 1.5 g. This indicates that higher particle concentrations result in stronger scattering, producing more pronounced enhancement of the optical signal. In both cases, multiband responses remained consistent, with the ranking of white > green > red > blue, and white and green channels showing the greatest amplitude. This further confirms that these wavelengths are the most sensitive to changes in suspension turbidity. From a mechanistic perspective, clay particles, owing to their smaller size, higher specific surface area, and greater suspension stability, were more likely to form uniformly distributed colloidal systems at high concentrations. This led to rapid intensification of scattering and multiple scattering effects, whereby a large proportion of scattered light was redirected and re-coupled into the fiber, causing a rapid increase in light intensity that remained at a high stable level. Compared with the silica system, clay suspensions at high concentrations produced smoother and more stable curves without pronounced fluctuations or declines, suggesting that particle distributions were more uniform and that aggregation and sedimentation effects had less interference on sensor response.

A comparison of Figure 7 and Figure 8 reveals the progressive evolution of clay suspension responses with increasing concentration. At low concentrations, the 0.5 g case showed a stepwise rising curve, indicating intermittent particle arrival at the probe location, whereas the 1.0 g case transitioned to a smoother curve with a faster rise and higher plateau. At higher concentrations, 1.5 g and 2.0 g suspensions showed rapid increases in light intensity within short times, followed by stable plateaus. The overall curves were smooth, and plateau values rose significantly with concentration. Thus, with increasing concentration, the distribution of clay particles evolved from intermittent to continuous, and sensor responses shifted from stepwise to smooth rapid rise and stabilization. The optical mechanism stems primarily from the high dispersibility and strong scattering capacity of fine clay particles in water, where multiple scattering effects quickly dominate at high concentrations, ultimately producing stable high-intensity plateaus.

By comparing the RR-POF monitoring results for silica powder and clay suspensions (Figure 5, Figure 6, Figure 7 and Figure 8), commonalities and differences in sensor responses under varying material and concentration conditions were identified. For both silica and clay suspensions, increasing concentration led to a rapid rise in light intensity followed by stabilization. Higher concentrations resulted in faster rise rates and higher plateau values, reflecting enhanced scattering due to higher particle densities. At low concentrations, stepwise rises were observed, indicating intermittent passage of aggregates or sedimentation fronts. At higher concentrations, particle distributions became more continuous, and the curves exhibited smooth, monotonic growth. In high-concentration silica suspensions, fluctuations or slight declines after peak values were common, caused by particle aggregation, sedimentation, and absorption effects, which dynamically altered local turbidity fields. In contrast, clay suspensions maintained smoother, more stable curves, due to their smaller particle size, larger specific surface area, and stable colloidal dispersion. Silica suspensions were more influenced by gravity-driven processes, whereas clay suspensions exhibited more colloidal stability. Across all experimental conditions, the ranking consistently followed this order: white had the highest sensitivity, followed by green, red, and blue. The white and green bands showed the highest sensitivity and amplitude, making them more suitable for quantitative monitoring, while red and blue exhibited relatively weaker responses due to material loss and lower source power. This confirms that the RR-POF sensor maintained good stability and consistency across wavelengths, enabling multiparameter analysis based on wavelength sensitivity.

Overall, concentration effects were consistently observed for both particle types, but particle characteristics led to distinct differences: silica suspensions exhibited more complex dynamics with stepwise, smooth, and fluctuating behaviors, while clay suspensions demonstrated a monotonic evolution from stepwise to smooth curves with higher stability. These findings not only highlight the influence of particle physical properties on optical monitoring patterns but also provide experimental evidence for the application of RR-POF sensors in diverse particulate systems.

### 3.2. Gap-POF Turbidity Monitoring

#### 3.2.1. Monitoring of Silica Powder Suspensions by Gap-POF

**Figure 9:**
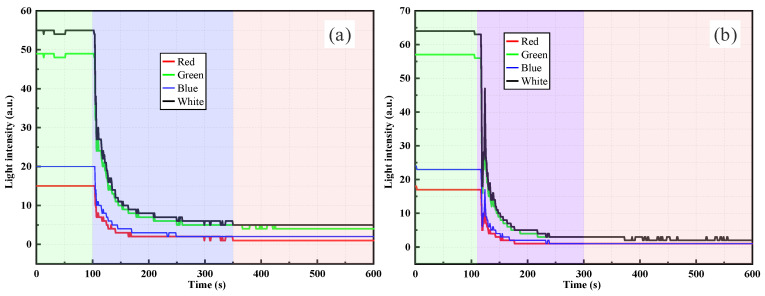
Gap-POF monitoring results for silica suspensions: (**a**) 0.5 g dry weight, (**b**) 1.0 g dry weight.

Figure 9 presents the light intensity monitoring results of the Gap-POF sensor in silica powder suspensions with different concentrations. The Gap-POF sensor transmits light signals across a 10 mm gap between two optical fibers. After suspension injection, a significant attenuation of light intensity was observed. In Figure 9a, when 0.5 g of silica powder suspension was introduced, the light intensity rapidly decreased and eventually stabilized, indicating that particles entering the gap region strongly impeded light transmission. Under low concentration conditions, the limited number of particles led to a more gradual attenuation, with the signal finally stabilizing at a relatively higher level. In contrast, as shown in Figure 9b, the 1.0 g suspension produced a sharper decline and reached a lower plateau, suggesting that the increased number of silica particles enhanced both scattering and absorption effects, thus accelerating light attenuation. Multiband monitoring demonstrated that white and green light were more sensitive to turbidity variations, whereas red and blue exhibited weaker responses, highlighting the wavelength-dependent transmission characteristics of light in suspensions. Overall, the Gap-POF sensor effectively reflected concentration changes via scattering and absorption, and under higher concentrations, produced more pronounced attenuation effects.

**Figure 10:**
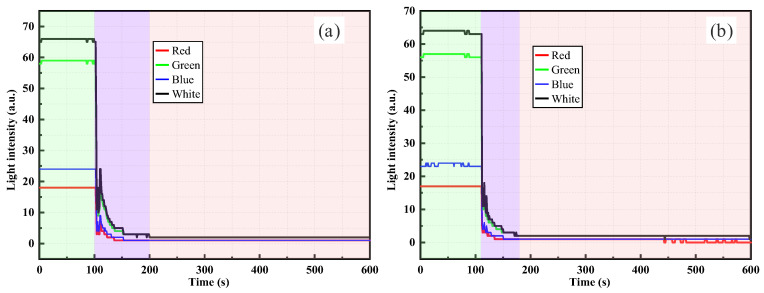
Gap-POF monitoring results for silica suspensions: (**a**) 1.5 g dry weight, (**b**) 2.0 g dry weight.

Figure 10 shows the light intensity responses of the Gap-POF sensor in higher-concentration silica suspensions. In Figure 10a, under the 1.5 g condition, light intensity decreased rapidly after injection and then gradually stabilized at a relatively low level. At lower concentrations, attenuation was slower since fewer particles entered the gap, resulting in weaker blocking effects. By contrast, in Figure 10b, the 2.0 g suspension exhibited a more substantial attenuation, where a larger number of particles in the gap caused stronger blocking of the optical path, leading to a rapid decrease and stabilization at an even lower plateau. The multiband results consistently showed that white and green channels had the strongest responses, while red and blue were weaker. This reflects both the wavelength-dependent losses of polymer fibers and particle scattering effects. Overall, with increasing particle concentration, signal attenuation intensified significantly, demonstrating the high sensitivity of the Gap-POF sensor to turbidity changes.

Compared with Figure 9, Figure 10 illustrates the stronger attenuation effects of higher concentrations. In the lower concentration cases of Figure 8, attenuation was relatively gradual, and light intensity eventually reached stable values. In contrast, the higher concentration suspensions in Figure 10 produced sharper declines, particularly in the 2.0 g case, where the amplitude of attenuation was most pronounced. This clearly indicates that under the operational principle of the Gap-POF sensor, increasing particle concentration significantly amplifies the blocking effect on light transmission, resulting in steeper intensity drops and lower stabilization levels. Hence, the Gap-POF sensor exhibits higher sensitivity in monitoring high-concentration suspensions, effectively capturing turbidity variations through pronounced attenuation responses.

#### 3.2.2. Monitoring of Clay Particle Suspensions by Gap-POF

**Figure 11:**
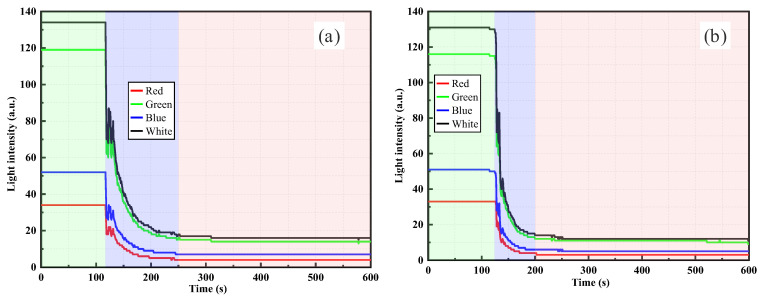
Gap-POF monitoring results for clay suspensions: (**a**) 0.5 g dry weight, (**b**) 1.0 g dry weight.

Figure 11 shows the light intensity responses of the Gap-POF sensor in clay suspensions with different concentrations. In Figure 11a, after injecting 0.5 g of clay suspension, the light intensity rapidly decreased and eventually stabilized. The initial signal remained at a relatively high level (approximately 120 a.u.), but as clay particles entered the gap region, light propagation was increasingly obstructed, leading to significant attenuation and stabilization at a lower plateau. Under low concentrations, the limited number of particles caused a gentler attenuation process. Compared with the silica suspensions shown in Figure 9 and Figure 10, clay particles, which have smaller particle sizes and better dispersibility in water, as evidenced by the particle size distribution curve in Figure 4, exhibited less optical attenuation at low concentrations.

In Figure 11b, the 1.0 g clay suspension produced greater attenuation, indicating that a higher concentration of particles significantly increased the blocking effect on light propagation. Following the initial sharp decrease after injection, the intensity stabilized at a lower value, suggesting that scattering and absorption effects were more pronounced at higher concentrations. The multiband results revealed that white and green light provided the strongest responses, highlighting their higher sensitivity to turbidity changes, whereas red and blue light exhibited weaker responses due to their longer wavelengths being more susceptible to scattering and absorption.

**Figure 12:**
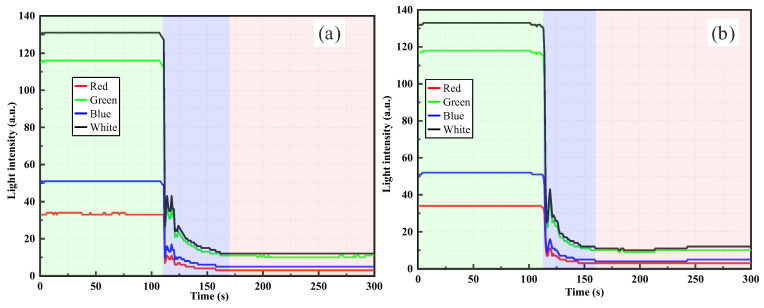
Gap-POF monitoring results for clay suspensions: (**a**) 1.5 g dry weight, (**b**) 2.0 g dry weight.

Figure 12 further illustrates the Gap-POF responses under different clay concentrations. In Figure 12a, the 1.5 g suspension resulted in a rapid decrease in intensity followed by stabilization at a low plateau. At lower concentrations, fewer particles weakened the blocking effect, producing smoother attenuation. In contrast, Figure 12b shows the 2.0 g suspension, where attenuation was more severe, with a rapid drop followed by stabilization at an even lower value. This confirmed that with higher particle concentrations, scattering and absorption effects became stronger, leading to faster signal decay. The multiband responses were consistent with earlier findings, with white and green light showing the most significant sensitivity to turbidity variations, while red and blue remained weaker.

Compared with the lower concentration cases in Figure 11, the higher concentrations in Figure 12 demonstrated more severe attenuation, particularly in the 2.0 g case, where the drop in intensity was largest. While attenuation in Figure 11 was relatively gradual, the high concentration suspensions in Figure 12 produced sharp declines and stabilization at much lower levels, highlighting the stronger effects of particle blocking at higher concentrations. In addition, compared with the results of silica suspensions in Figure 9 and Figure 10, the light intensity attenuation in clay suspensions at high concentrations appeared smoother and exhibited a more linear trend. In silica suspensions, higher concentrations often led to stronger fluctuations and irregular variations in intensity. This is attributed to the larger particle size and poorer dispersibility of silica in water, which tended to cause local aggregation in the gap region, resulting in unstable scattering and absorption effects. In contrast, clay particles, with a smaller size, larger specific surface area, and better water dispersibility, were more evenly distributed within the suspension. This uniform dispersion produced more continuous, stable, and predictable attenuation of light intensity. The difference in particle size between silica powder and clay significantly affects light scattering behavior. Larger silica particles induce more irregular fluctuations due to local aggregation, while smaller clay particles generate smoother attenuation curves owing to better dispersion and stronger multiple scattering.

### 3.3. Response Time and Sensitivity Analysis of POF Sensors Under Different Concentrations

In the previous sections, we discussed the various factors affecting the performance of the POF sensors, including their sensitivity and response to changes in turbidity under different conditions. To further analyze the sensor’ s ability to detect turbidity changes, we now define and examine the concept of response time, which plays a crucial role in evaluating the sensor’s performance. The response time *t*_90_ is defined as the time required for the optical intensity signal (*I*(*t*)) to reach 90% of the total change between its initial value (*I*_0_) and final steady-state value (*I_s_*).The statistical results are shown in Table 1.

By comparing the monitoring results of RR-POF and Gap-POF sensors in silica powder and clay suspensions (see Table 1), we can summarize the effects of concentration on the response time of the sensors and highlight the performance differences between the two sensor configurations. At low concentrations, the RR-POF sensor exhibited fluctuations in response time, indicating that at low concentrations, the light intensity changes were slower, and the sensor responded less sensitively to minor changes. As the concentration increased, both silica powder and clay suspensions showed a gradual decrease in response time. This suggests that in high-concentration suspensions, the increased light scattering and particle density allow the sensor to respond more sensitively to turbidity changes, resulting in smoother curves and more stable response times. The behavior of RR-POF makes it particularly suitable for long-term monitoring in high-concentration scenarios, such as industrial wastewater or construction runoff, where continuous and stable turbidity monitoring is needed. In contrast, the Gap-POF sensor showed generally shorter response times compared to RR-POF and exhibited a rapid decrease in response time with increasing concentration. In low-concentration suspensions, the sensor responded quickly, effectively capturing turbidity changes. However, as the concentration increased, Gap-POF’s response time decreased rapidly, leading to a loss of accuracy at higher concentrations. This feature reflects the Gap-POF’s higher sensitivity and faster response, but also highlights its limitation in terms of precision at high turbidity levels. Gap-POF is better suited for rapid monitoring in low-concentration scenarios, particularly in applications where fast response times are critical, such as in real-time water quality monitoring or during the casting of cement.

**Figure 13:**
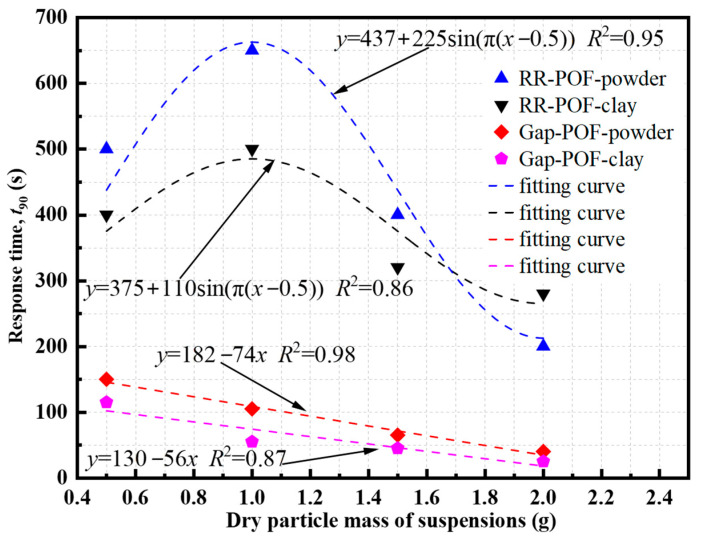
Response time variation in RR-POF and Gap-POF sensors with different suspension concentration.

As shown in Figure 13, to further quantify the dynamic response characteristics of both POF sensor types, the response time with dry particle mass for the RR-POF and Gap-POF sensors in both silica powder and clay suspensions. For the RR-POF sensors, the response time exhibits a nonlinear oscillatory trend with increasing particle mass. Specifically, increases initially, reaching a maximum at approximately 1.0 g, followed by a gradual decline at higher particle masses. This behavior can be well represented by a sinusoidal fitting equation y=437+225sin(π(x-0.5)) for silica powder suspensions (*R*^2^ = 0.95), and  y=375+110sin(π(x−0.5)) for clay suspensions (*R*^2^ = 0.86). The initial increase in response time results from enhanced multiple scattering and particle interactions, which delay the stabilization of the optical signal. At higher concentrations, however, particle clustering and light-path saturation lead to faster signal convergence, thus reducing *t*_90._

In contrast, the Gap-POF sensors demonstrate a nearly linear decrease in response time with increasing particle concentration, following *y* = 182 − 74*x* (*R*^2^ = 0.98) for silica powder and y = 130 − 56x (*R*^2^ = 0.87) for clay suspensions. This trend indicates that the gap-type configuration facilitates more direct light attenuation through scattering and absorption, allowing faster stabilization as particle density increases. The enhanced light-particle coupling within the gap accelerates the attainment of equilibrium optical intensity and reflects superior dynamic responsiveness under turbid conditions. Overall, the quantitative fitting analysis confirms that the RR-POF sensor exhibits a nonlinear concentration-dependent response time governed by refraction and reflection coupling as well as transient scattering behavior, whereas the Gap-POF sensor shows a monotonic acceleration of response with increasing turbidity dominated by direct transmission attenuation. These differences illustrate the distinct sensing mechanisms of the two optical fiber configurations.

Building upon the comparison, a more detailed evaluation highlights the distinct advantages of each sensor configuration for specific turbidity monitoring needs. The Gap-POF sensor, due to its design, experiences a sharp decrease in light intensity when particles obstruct the light transmission path, making it highly sensitive. This characteristic makes it particularly well-suited for rapid turbidity detection in dynamic environments where quick response is crucial. For example, in applications like cement hydration monitoring during the casting of concrete, where turbidity changes occur rapidly at preset monitoring points, the Gap-POF sensor can effectively capture these transient changes in real-time. In contrast, the RR-POF sensor, based on total internal reflection, provides a more stable response over time and excels in high-concentration, high-scattering environments. This stability makes it ideal for long-term monitoring applications, such as industrial wastewater or construction site runoff, where sustained accuracy over extended periods is necessary despite fluctuating turbidity levels. Together, these sensors provide a comprehensive solution for adaptive turbidity monitoring across a wide range of geotechnical and environmental applications.

## 4. Discussion

This study presents a promising application of POF sensors for turbidity monitoring, although several limitations require further investigation. Firstly, while the feasibility of the POF sensors has been demonstrated, the metrological characteristics such as accuracy, repeatability, and long-term stability were only partially evaluated. Although repeatability tests and sensitivity analyses were conducted, additional verification under varying environmental conditions is needed. Baseline drift may also affect long-term measurements, and to address this, an automatic baseline calibration method using a reference channel is planned for future designs. Another aspect requiring further exploration concerns the optical scattering and absorption properties of suspended particles. Although basic approximations for silica and clay suspensions were discussed, more quantitative measurements of particle refractive indices and absorption spectra are needed, particularly for heterogeneous mixtures with complex compositions. These parameters will support improved sensor calibration and enhance the accuracy of turbidity quantification. In addition, the spectral loss characteristics of the polymer optical fiber were not measured in the present study. This is mainly because the sensing system was developed as an economical and easily deployable prototype based on a broadband LED and low-cost POF. As a consequence, wavelength-dependent attenuation was not examined. It is recognized that such attenuation may influence multispectral measurements, and a more detailed evaluation of fiber loss behavior will be carried out in future work to refine sensor calibration and improve measurement accuracy. Mechanical degradation of fiber ends in flowing water remains another important consideration. Since all experiments in the present study were conducted under static laboratory conditions, the effects of water flow on signal stability and potential abrasion of the exposed fiber tip were not captured. In practical environments, moving water and suspended particles may introduce additional scattering fluctuations and gradually erode the fiber end surface. Future work will therefore examine the influence of flow-induced disturbances and will incorporate protective coatings or housing structures to enhance long-term durability under dynamic hydraulic conditions. Finally, although multispectral measurements were used in this study, their primary purpose was to explore the wavelength-dependent behavior of turbidity response and to identify the most sensitive spectral regions. The comparative analysis of white, green, red, and blue channels provided useful insight into the relative performance of different wavelengths and confirmed that white and green light exhibited the strongest sensitivity. These findings offer guidance for future sensor optimization. In practical field deployment, the system can be simplified by selecting a single optimal wavelength to reduce instrument complexity while retaining sufficient measurement accuracy.

## 5. Conclusions

This study employed two types of POF sensors to visualize turbidity variations in suspensions with different particle concentrations and compositions. The main findings can be summarized as follows:

(1) For both RR-POF and Gap-POF sensors, higher particle concentrations led to greater attenuation of light intensity, with final stabilization at lower plateau levels. As concentration increased, scattering and absorption effects became more pronounced, producing faster signal decay and lower stable intensities. These results validate the feasibility of using POF sensors for visualized monitoring in geotechnical and environmental engineering.

(2) Distinct differences were observed between silica and clay particles. At high concentrations, silica suspensions induced irregular fluctuations in intensity, whereas clay suspensions, owing to their smaller particle size and better dispersion, produced smoother and more uniform attenuation trends. This indicates that the Gap-POF sensor exhibits different response characteristics depending on particle composition.

(3) Across all experimental conditions, white and green light exhibited the strongest responses, demonstrating their higher sensitivity to turbidity variations. Red and blue light showed weaker responses, suggesting that longer wavelengths are more strongly affected by scattering and absorption. Thus, white and green light signals can serve as key indicators for turbidity monitoring.

(4) The Gap-POF sensor is ideal for low-concentration ranges and applications requiring rapid turbidity detection, providing quick responses to slight changes in light intensity. In contrast, the RR-POF sensor excels in high-concentration suspensions and is better suited for long-term monitoring, offering stable readings under high turbidity conditions for sustained accuracy over time.

## Figures and Tables

**Table 1:** Response time of POF sensors under different concentrations.

Particle Dry Weight (g)	Soil	Sensor Type	Response Direction	*t*_90_ (s)
0.5	Silica powder	RR-POF	Increasing	500
1.0	Silica powder	RR-POF	Increasing	650
1.5	Silica powder	RR-POF	Increasing	400
2.0	Silica powder	RR-POF	Increasing	200
0.5	DL clay	RR-POF	Increasing	400
1.0	DL clay	RR-POF	Increasing	500
1.5	DL clay	RR-POF	Increasing	320
2.0	DL clay	RR-POF	Increasing	280
0.5	Silica powder	Gap-POF	Decreasing	150
1.0	Silica powder	Gap-POF	Decreasing	105
1.5	Silica powder	Gap-POF	Decreasing	65
2.0	Silica powder	Gap-POF	Decreasing	40
0.5	DL clay	Gap-POF	Decreasing	115
1.0	DL clay	Gap-POF	Decreasing	55
1.5	DL clay	Gap-POF	Decreasing	45
2.0	DL clay	Gap-POF	Decreasing	25

## Data Availability

The original contributions presented in this study are included in the article. Further inquiries can be directed to the corresponding author.
